# Composite cardiovascular health indices (Life’s essential 8 and Life’s crucial 9) and female infertility: an NHANES 2013–2018 cross−sectional analysis

**DOI:** 10.3389/fendo.2025.1581148

**Published:** 2025-11-28

**Authors:** Yukun Duan, Peixiu Liu, Hui Li, Yanping Gao

**Affiliations:** Department of Obstetrics and Gynecology, The First People’s Hospital of Datong, Datong, China

**Keywords:** female infertility, cardiovascular health, life's essential 8, life's crucial 9, insulin resistance, inflammation, reproductive function

## Abstract

**Background:**

Female infertility affects 10–15% of couples worldwide and is influenced by multiple factors, including cardiovascular and metabolic health. This study examines the association between composite cardiovascular health indices—Life’s Essential 8 (LE8) and Life’s Crucial 9(LC9)—and the risk of infertility.

**Methods:**

We conducted a cross-sectional analysis of NHANES 2013–2018 data on 2,360 women aged 20–45 years, incorporating the NHANES complex survey design weights. We used survey−weighted multivariable logistic regression, restricted cubic splines (RCS), and subgroup/interaction analyses. For prediction, we applied LASSO with 10−fold cross−validation, followed by multivariable logistic regression to construct a nomogram. Discrimination (AUC with 95% CI), bootstrap calibration (1,000 resamples), and decision curve analysis (DCA) were reported.

**Results:**

In fully adjusted models, women in the highest quartile of LE8 had lower odds of infertility than those in the lowest quartile (OR 0.39, 95% CI 0.26–0.58), and similarly for LC9 (OR 0.43, 95% CI 0.29–0.65; p−trend < 0.001). The prediction nomogram showed moderate discrimination (AUC 0.691, 95% CI 0.668–0.714) with good internal calibration; no external validation was performed.

**Conclusion:**

Better composite cardiovascular health—captured by LE8 and LC9—is associated with lower prevalence of self−reported infertility. This cross-sectional design precludes causal inference. Given the nomogram’s moderate AUC and lack of external validation, the model’s clinical utility is limited. Prospective studies are warranted.

## Introduction

Infertility is a significant public health concern, affecting approximately 10–15% of couples worldwide (1, 2). It is defined as the inability to achieve a clinical pregnancy after 12 months of regular, unprotected sexual intercourse (3, 4). The complex interplay of genetic, physiological, environmental, and lifestyle factors influences this process (5, 6). Infertility is often categorized into primary infertility, where a woman has never conceived, and secondary infertility, where a woman has previously conceived but is unable to conceive again (7). The consequences of infertility extend beyond the individual level, influencing family dynamics, societal structures, and healthcare systems. Despite advances in reproductive medicine, infertility remains a significant burden, with many cases lacking clear etiological explanations. Understanding modifiable risk factors associated with infertility is crucial for developing effective prevention and intervention strategies.

Among the numerous factors contributing to infertility, lifestyle behaviors and overall health status have gained increasing attention in recent years (8). Diet, physical activity, obesity, metabolic health, and psychosocial stress have all been implicated in reproductive function (9, 10). The American Heart Association (AHA) developed Life’s Essential 8 (LE8) as a comprehensive measure of cardiovascular health, incorporating key behaviors such as diet quality, physical activity, nicotine exposure, sleep health, and biological factors, including body mass index (BMI), blood pressure, blood glucose, and blood lipid levels (11). More recently, Life’s Crucial 9 (LC9) was introduced as an extension of LE8, integrating psychological well-being to provide a more comprehensive health assessment (12). Both indices are associated with cardiovascular disease, metabolic syndrome, and all-cause mortality, but their potential role in reproductive health and infertility risk remains largely unexplored (13, 14). LE8 and LC9 are composite 0–100 indices, where higher scores indicate better cardiovascular and overall health. LC9 includes all eight LE8 components plus a psychological well-being component, offering a broader construct than LE8.

Growing evidence suggests that metabolic health and cardiovascular fitness are closely linked to reproductive outcomes. Poor diet, obesity, insulin resistance, and chronic inflammation—key factors captured by LE8 and LC9—have been implicated in ovulatory dysfunction, polycystic ovary syndrome (PCOS), endometrial receptivity disorders, and impaired gamete quality (15). Chronic low-grade inflammation, often driven by poor dietary habits and metabolic dysfunction, has been found to alter hormonal balance, leading to anovulation and luteal phase defects (16). Furthermore, increased adiposity, particularly visceral fat accumulation, is strongly associated with hyperinsulinemia and elevated androgen levels, key features of PCOS that contribute to infertility (17). Psychological stress and mental health, as measured by LC9, have been shown to disrupt hypothalamic–pituitary–gonadal (HPG) axis function, contributing to menstrual irregularities and infertility (18). Stress-induced activation of the hypothalamic–pituitary–adrenal (HPA) axis results in elevated cortisol levels, which can impair gonadotropin-releasing hormone (GnRH) pulsatility and reduce luteinizing hormone (LH) and follicle–stimulating hormone (FSH) secretion, thereby affecting ovulation (19). Additionally, mental health disorders such as depression and anxiety have been linked to alterations in immune function and increased oxidative stress, both of which can negatively impact reproductive outcomes (20).

The present study leverages data from the National Health and Nutrition Examination Survey (NHANES) 2013-2018 to explore the associations between LE8, LC9, and infertility among women of reproductive age (21). We employ multivariable logistic regression models to assess the independent effects of these indices on infertility while adjusting for potential confounders, including socioeconomic status, comorbidities, and lifestyle factors. Additionally, we conducted subgroup analyses to examine effect modifications by demographic and lifestyle factors, including age, BMI, and smoking status. Furthermore, we utilize the least absolute shrinkage and selection operator (LASSO) regression and machine learning-based approaches to develop predictive models for infertility based on LE8 and LC9. We hypothesized that higher LE8 and LC9 scores are independently associated with lower odds of infertility and that these indices improve risk prediction beyond conventional covariates (age, BMI, smoking). We aimed to (i) estimate associations using survey−weighted models, (ii) assess dose–response with RCS, {(iii) evaluate effect modification (age, BMI, smoking, hypertension), and (iv) develop and internally validate a LASSO−selected nomogram.

This study aims to address an important knowledge gap in reproductive epidemiology by investigating the role of composite health indices in infertility risk. The findings from this research may inform targeted public health interventions and lifestyle modification programs aimed at improving fertility outcomes through comprehensive health management. Additionally, identifying key components of LE8 and LC9 that exert the most decisive influence on infertility could pave the way for future mechanistic studies and precision medicine approaches in reproductive health. Ultimately, this study highlights the importance of an integrative health assessment framework that encompasses both metabolic and psychological dimensions in reproductive health research. We hypothesized that higher LE8 and LC9 scores are independently associated with lower odds of infertility and that these indices provide incremental predictive value beyond conventional factors (age, BMI, smoking).

## Materials and methods

### Study design and data source

This cross-sectional study utilized data from the NHANES 2013–2018, a nationally representative survey administered by the Centers for Disease Control and Prevention (CDC). The NHANES employs a complex, multistage probability sampling design to collect health and nutrition data from the U.S. civilian, noninstitutionalized population. Stratified multistage probability sampling was used to mitigate the bias resulting from post-stratification, non-response, and oversampling. A specific sampling weight was assigned to each participant to ensure nationwide representativeness. The detailed survey protocols and data collection methodologies used are publicly available on the NHANES website. We analyzed the 2013–2018 cycles using the NHANES complex survey design with strata (SDMVSTRA), primary sampling units (SDMVPSU), and combined 6-year examination weights per CDC guidance. For MEC-exam variables, we used WTMEC2YR and derived 6-year weights as WTMEC2YR/3. All analyses-including descriptive statistics, multivariable logistic regression, restricted cubic splines (RCS), ROC/AUC, calibration, and decision curve analysis (DCA)-were performed within the survey design framework, applying strata, PSUs, and the combined weights.

### Study population

**Figure 1:**
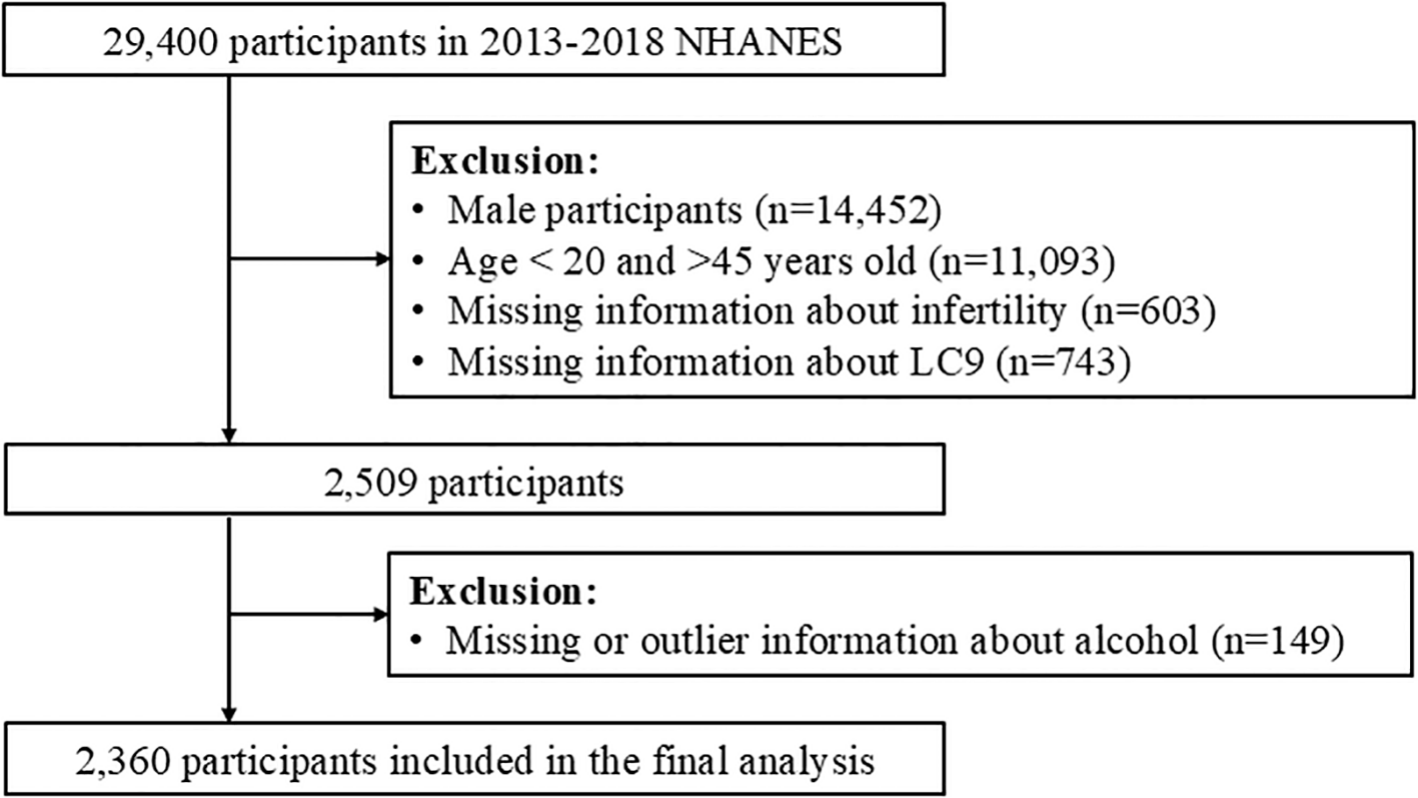
Flowchart of participant selection from NHANES 2013–2018, showing inclusion/exclusion steps and the final analytic sample (n=2,360 women aged 20–45).

From the NHANES dataset, a total of 29,400 participants were initially considered. We applied the following exclusion criteria (1): male participants (n=14,452) (2), individuals younger than 20 years or older than 45 years (n=11,093) (3), those with missing data on infertility status (n=603), and (4) participants with missing or outlier data for the LC9 or LE8 components (n=743). After these exclusions, a final analytic sample of 2,360 women was included in the study (**Figure 1**).

### Exposure variables

The primary exposure variables were Life’s Essential 8 (LE8) and Life’s Crucial 9 (LC9) scores. LE8 scores were calculated based on eight key health metrics: diet, physical activity, nicotine exposure, sleep health, BMI, blood pressure, blood glucose, and blood lipid levels. LC9 included all LE8 components along with an additional component assessing psychological well-being. Both indices were categorized into quartiles for analysis. For LC9, psychological well−being was assessed using the Patient Health Questionnaire-9 (PHQ-9; range 0-27, higher scores indicate worse depressive symptoms). We reverse-coded the PHQ-9 and linearly rescaled it to 0-100 so that higher LC9 indicates better well−being, using the formula: Well-being(0–100)=(27-PHQ9_t_otal)/27×100. This approach is consistent with published applications of LC9 in NHANES analyses. Primary analyses employed quartiles (for interpretability and robustness to non-linearity), with sensitivity analyses modeling LE8/LC9 as continuous variables via RCS.

### Outcome variable

Infertility was defined based on self-reported responses to NHANES reproductive health questionnaires. Participants were classified as infertile if they reported attempting conception for ≥12 months without success.

### Covariates

Demographic and lifestyle factors were included as covariates: age, race/ethnicity, education level, marital status, income–poverty ratio, alcohol consumption, smoking status, BMI category, hypertension status, and diabetes status. Marital status was categorized according to NHANES categories: married/living with a partner, divorced/separated/widowed, and never married; in descriptive analyses, unmarried refers to the latter two groups. The poverty-income ratio (PIR) was categorized as follows: < 1.3, 1.3–<3.5, and ≥3.5.

### Statistical analysis

Descriptive statistics were used to summarize the baseline characteristics. Continuous variables are presented as weighted means with 95% confidence intervals (CIs), whereas categorical variables are expressed as proportions with 95% CIs. Differences between the infertile and noninfertile groups were assessed via the adjusted Wald test for continuous variables and the Rao–Scott chi-square test for categorical variables. Spearman correlation analysis was used to evaluate the relationships among LE8, LC9, and infertility.

Weighted multivariable logistic regression was applied to examine the associations among LE8, LC9, and infertility, adjusting for potential confounders. Subgroup analyses stratified by age, BMI, smoking status, and socioeconomic status were conducted, and the results were visualized via forest plots. We also employed restricted cubic splines to explore potential nonlinear relationships between LE8, LC9, and infertility.

For predictive modeling, we utilized least absolute shrinkage and selection operator (LASSO) regression for variable selection, followed by machine learning-based predictive modeling. A nomogram model was constructed, and its discriminatory power was evaluated via receiver operating characteristic (ROC) curves and the area under the curve (AUC). Model calibration was assessed via calibration plots, and decision curve analysis was conducted to evaluate clinical utility. Sensitivity analyses, including multiple imputations for missing data, were performed to assess the robustness of our findings. Sensitivity analyses assessed robustness across alternate specifications (e.g., modeling LE8/LC9 as continuous with RCS, subgroup/interaction analyses). We conducted complete−case analyses; no multiple imputation was performed.

## Results

### Baseline characteristics

Of 2,360 participants, 285 (12.08%) had infertility (**Table 1**). Compared with the noninfertility group, the infertility group was older (34.87 ± 7.10 vs 32.53 ± 7.62 years; P < 0.001), had higher SBP (115.44 ± 12.42vs 113.30 ± 13.04 mmHg; P = 0.009) with a nonsignificant DBP trend (P = 0.057), and included more individuals aged ≥40 years (14.93% vs 8.38%; P < 0.001). Race/ethnicity and education were not associated with infertility. Marital status differed (married/cohabiting: 15.47% vs never married: 5.97%; P < 0.001). BMI was significantly associated with the highest prevalence at ≥30kg/m² (15.85%; P < 0.001).

**Table 1:** Baseline characteristics of participants from NHANES 2013–2018.

Characteristic	Overall (n=2360)	No infertility (n=2075)	Infertility (n=285)	P-value
Age	32.81 ± 7.60	32.53 ± 7.62	34.87 ± 7.10	**<0.001**
Mean SBP (mmHg)	113.56 ± 12.99	113.30 ± 13.04	115.44 ± 12.42	**0.009**
Mean DBP (mmHg)	68.89 ± 10.65	68.73 ± 10.58	70.01 ± 11.12	0.057
Age strata, n (%)				**<0.001**
<30	883 (37.42)	809 (91.62)	74 (8.38)	
30-39	881 (37.33)	759 (86.15)	122 (13.85)	
≥40	596 (25.25)	507 (85.07)	89 (14.93)	
Race, n (%)				0.141
Mexican American	405 (17.16)	359 (88.64)	46 (11.36)	
Other Hispanic	247 (10.47)	226 (91.50)	21 (8.50)	
Non-Hispanic White	819 (34.70)	703 (85.84)	116 (14.16)	
Non-Hispanic Black	512 (21.69)	452 (88.28)	60 (11.72)	
Other Race	377 (15.97)	335 (88.86)	42 (11.14)	
Education level, n (%)				0.738
Less than 9th grade	107 (4.53)	98 (91.59)	9 (8.41)	
9-11th grade	250 (10.59)	220 (88.00)	30 (12.00)	
High school graduate	442 (18.73)	384 (86.88)	58 (13.12)	
Some college or associates degree	891 (37.75)	781 (87.65)	110 (12.35)	
College graduate or above	670 (28.39)	592 (88.36)	78 (11.64)	
Marital status, n (%)				**<0.001**
Married/Living with a partner	1377 (58.35)	1164 (84.53)	213 (15.47)	
Divorced/Separated/Widowed	263 (11.14)	234 (88.97)	29 (11.03)	
Never married	720 (30.51)	677 (94.03)	43 (5.97)	
BMI, n (%)				**<0.001**
<25 kg/m2	764 (32.37)	687 (89.92)	77 (10.08)	
25-30 kg/m2	574 (24.32)	528 (91.99)	46 (8.01)	
≥30 kg/m2	1022 (43.31)	860 (84.15)	162 (15.85)	
Income-to-poverty ratio, n (%)				0.268
<1.3	778 (32.97)	695 (89.33)	83 (10.67)	
1.3-3.5	989 (41.91)	867 (87.66)	122 (12.34)	
≥3.5	593 (25.13)	513 (86.51)	80 (13.49)	
Alcohol use, n (%)				**0.008**
Yes	1159 (49.11)	998 (86.11)	161 (13.89)	
No	1201 (50.89)	1077 (89.68)	124 (10.32)	
Smoking, n (%)				**0.010**
Yes	683 (28.94)	582 (85.21)	101 (14.79)	
No	1677 (71.06)	1493 (89.03)	184 (10.97)	
Hypertension, n (%)				**0.005**
Yes	423 (17.92)	355 (83.92)	68 (16.08)	
No	1937 (82.08)	1720 (88.80)	217 (11.20)	
Diabetes, n (%)				0.697
Yes	1341 (56.82)	1176 (87.70)	165 (12.30)	
No	1019 (43.18)	899 (88.22)	120 (11.78)	
LC9	62.23 ± 12.08	62.67 ± 12.07	59.03 ± 11.67	**<0.001**
Blood lipid score	79.00 ± 27.28	79.43 ± 27.06	75.86 ± 28.65	**0.048**
Body mass index score	56.97 ± 37.23	58.30 ± 36.84	47.26 ± 38.64	**<0.001**
Blood glucose score	90.34 ± 20.87	90.99 ± 20.25	85.65 ± 24.47	**<0.001**
Blood pressure score	85.88 ± 25.13	86.38 ± 24.89	82.28 ± 26.55	**0.014**
Diet score	37.17 ± 31.12	37.39 ± 31.22	35.58 ± 30.41	0.356
Depression score	10.74 ± 20.49	10.35 ± 20.15	13.60 ± 22.63	**0.022**
Nicotine exposure score	76.52 ± 38.74	77.26 ± 38.28	71.18 ± 41.62	**0.020**
Physical activity score	40.88 ± 47.99	41.05 ± 48.02	39.61 ± 47.81	0.636
Sleep health score	82.58 ± 24.85	82.91 ± 24.70	80.21 ± 25.88	0.085
LE8	68.67 ± 13.88	69.21 ± 13.83	64.70 ± 13.65	**<0.001**

Continuous variables are presented as survey−weighted means (95% CI); categorical variables as weighted proportions (95% CI).

Categorical variables: values are expressed as numbers (percentages).

SBP, systolic blood pressure; DBP, diastolic blood pressure; BMI, body mass index; LC9, Life’s Crucial 9; LE8, Life’s Essential 8.

Bold values indicates statistically significant results.

Lifestyle and health metrics also differed. Alcohol use (P = 0.008) and smoking (P = 0.010) were more common in the infertility group; hypertension was more prevalent (16.08% vs 11.20%; P = 0.005), whereas diabetes was not (P = 0.697). Composite scores were lower with infertility: Life’s Crucial 9 (LC9;59.03 ± 11.67vs 62.67 ± 12.07; P < 0.001), blood lipids (75.86 ± 28.65vs 79.43 ± 27.06; P = 0.048), blood glucose (85.65 ± 24.47vs 90.99 ± 20.25; P < 0.001), and blood pressure (82.28 ± 26.55vs 86.38 ± 24.89; P = 0.014). Depression scores were higher (13.60 ± 22.63vs 10.35 ± 20.15; P = 0.022) and nicotine exposure scores lower (71.18 ± 41.62vs 77.26 ± 38.28; P = 0.020). The Life’s Essential 8 (LE8) score was also lower (64.70 ± 13.65vs 69.21 ± 13.83; P < 0.001). Diet (P = 0.356), physical activity (P = 0.636), and sleep health (P = 0.085) did not differ significantly.

### Associations among LE8, LC9, and infertility

The associations between LE8 and LC9 scores and the odds of infertility were assessed via three models: Model 1 (unadjusted), Model 2 (adjusted for age and race), and Model 3 (fully adjusted for age, race, education level, marital status, poverty-income ratio, and alcohol use).

For LE8, higher quartiles were significantly associated with lower odds of infertility across all the models. In Model 1, participants in Quartile 2 had a significantly lower odds ratio (OR) of infertility than did those in Quartile 1 (OR = 0.68, 95% CI: 0.49–0.94, P = 0.021), with further reductions observed in Quartile 3 (OR = 0.64, 95% CI: 0.46–0.90, P = 0.011) and Quartile 4 (OR = 0.39, 95% CI: 0.27–0.57, P < 0.001). A significant trend was observed across quartiles (p for trend < 0.001). Similar patterns were observed in Model 3, where Quartile 4 maintained a strong inverse association with infertility (OR = 0.39, 95% CI: 0.26–0.58, P < 0.001), and Quartile 2 and Quartile 3 remained protective (OR = 0.66, 95% CI: 0.47–0.93, P = 0.017; OR = 0.64, 95% CI: 0.45–0.91, P = 0.013, respectively).

For LC9, similar trends were observed, although the associations were slightly weaker than those for LE8. In Model 1, participants in Quartile 3 had reduced odds of infertility (OR = 0.66, 95% CI: 0.47–0.93, P = 0.016), and those in Quartile 4 had the strongest inverse association (OR = 0.42, 95% CI: 0.29–0.61, P < 0.001). In the fully adjusted Model 3, the inverse associations persisted, with Quartile 4 exhibiting the lowest odds of infertility (OR = 0.43, 95% CI: 0.29–0.65, P < 0.001). A significant trend was observed across quartiles for all the models (p for trend < 0.001) (**Table 2**).

**Table 2:** Association with LE8 and LC9 of the odds of infertility.

Characteristic		Model 1^a^		Model 2 ^b^		Model 3 ^c^	
		OR (95% CI)	P-value	OR (95% CI)	P-value	OR (95% CI)	P-value
LE8	Quartile 1	Ref		Ref		Ref	
	Quartile 2	0.68 (0.49, 0.94)	**0.021**	0.73 (0.52, 1.01)	0.060	0.66 (0.47, 0.93)	**0.017**
	Quartile 3	0.64 (0.46, 0.90)	**0.011**	0.71 (0.50, 1.00)	0.052	0.64 (0.45, 0.91)	**0.013**
	Quartile 4	0.39 (0.27, 0.57)	**<0.001**	0.44 (0.30, 0.64)	**<0.001**	0.39 (0.26, 0.58)	**<0.001**
	P for trend	**<0.001**		**<0.001**		**<0.001**	
LC9	Quartile 1	Ref		Ref		Ref	
	Quartile 2	0.81 (0.58, 1.12)	0.195	0.87 (0.63, 1.22)	0.425	0.81 (0.58, 1.14)	0.224
	Quartile 3	0.66 (0.47, 0.93)	**0.016**	0.74 (0.53, 1.04)	0.085	0.69 (0.49, 0.99)	**0.043**
	Quartile 4	0.42 (0.29, 0.61)	**<0.001**	0.47 (0.32, 0.70)	**<0.001**	0.43 (0.29, 0.65)	**<0.001**
	P for trend	**<0.001**		**<0.001**		**<0.001**	

OR: odds ratio.

95% CI: 95% confidence interval.

a Model 1: no covariates were adjusted.

b Model 2: adjusted for age and race.

c Model 3: adjusted for age, race, education level, marital status, PIR, alcohol use.

Bold values indicates statistically significant results.

### Subgroup analysis

**Figure 2:**
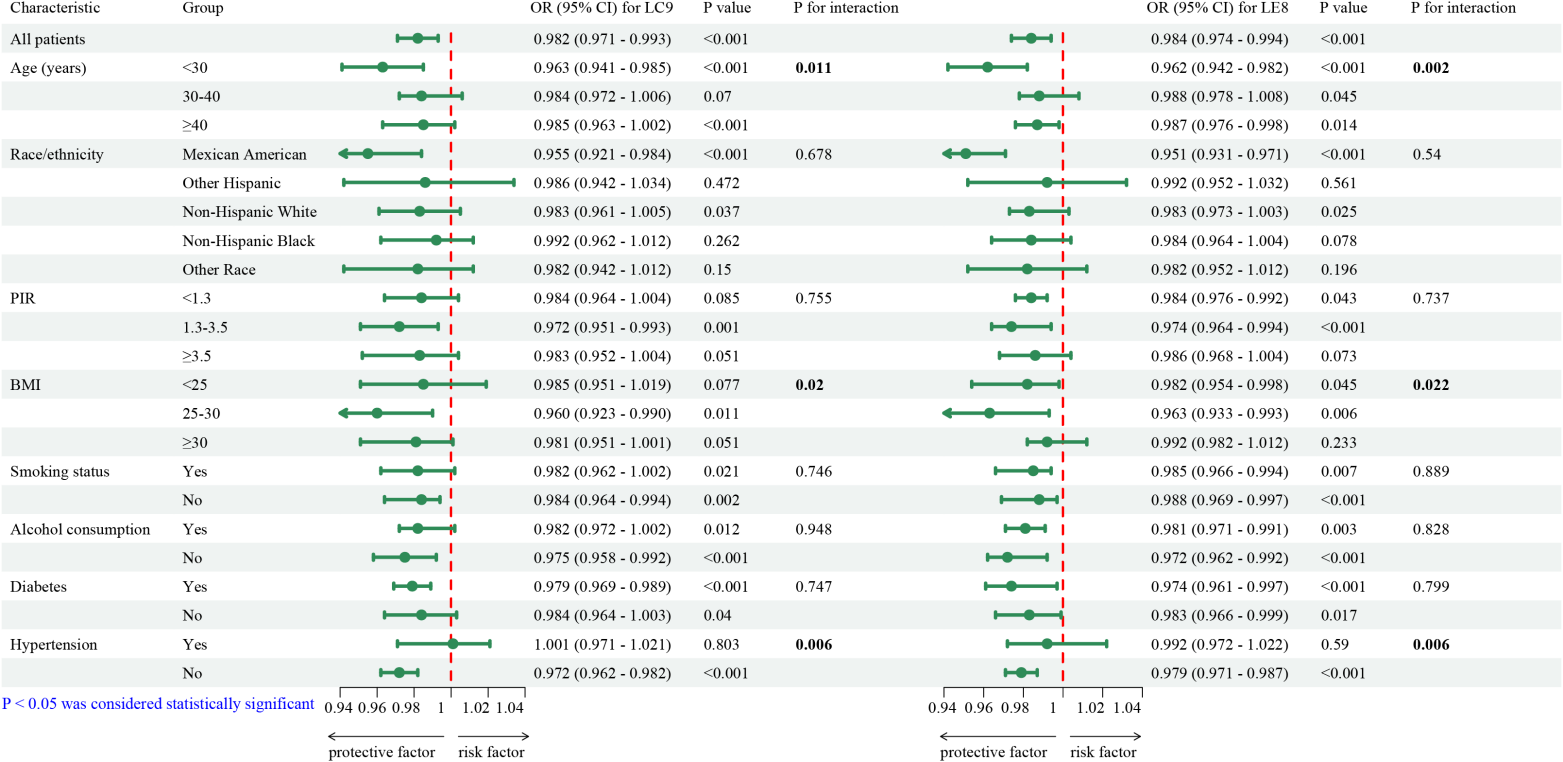
Survey-weighted, fully adjusted subgroup analyses of the association between LE8/LC9 and infertility. Forest plots show adjusted odds ratios (95% CIs) comparing highest vs lowest quartiles across subgroups (e.g., age, BMI, hypertension, smoking). Interaction p-values were assessed for each factor.

Subgroup analyses were conducted to examine effect modification by demographic and lifestyle factors. As shown in **Figure 2** (forest plot), the protective effects of LE8 and LC9 on infertility risk were more pronounced among younger women (<30 years) (OR = 0.963, 95% CI: 0.941–0.985, p < 0.011 for LE8; OR = 0.962, 95% CI: 0.942–0.982, p = 0.002 for LC9). Apart from age, the associations of LE8 and LC9 also showed significant differences across BMI and hypertension subgroups. The LE8 index exhibited a protective effect on BMI subgroups, except for those with a BMI between 25 and 30. Moreover, LC9 demonstrated a significant protective effect in the two BMI subgroups with a BMI less than 30. Additionally, both LE8 and LC9 exhibited substantial protective effects in individuals without hypertension.

### Nonlinear relationships among LE8, LC9, and infertility

**Figure 3:**
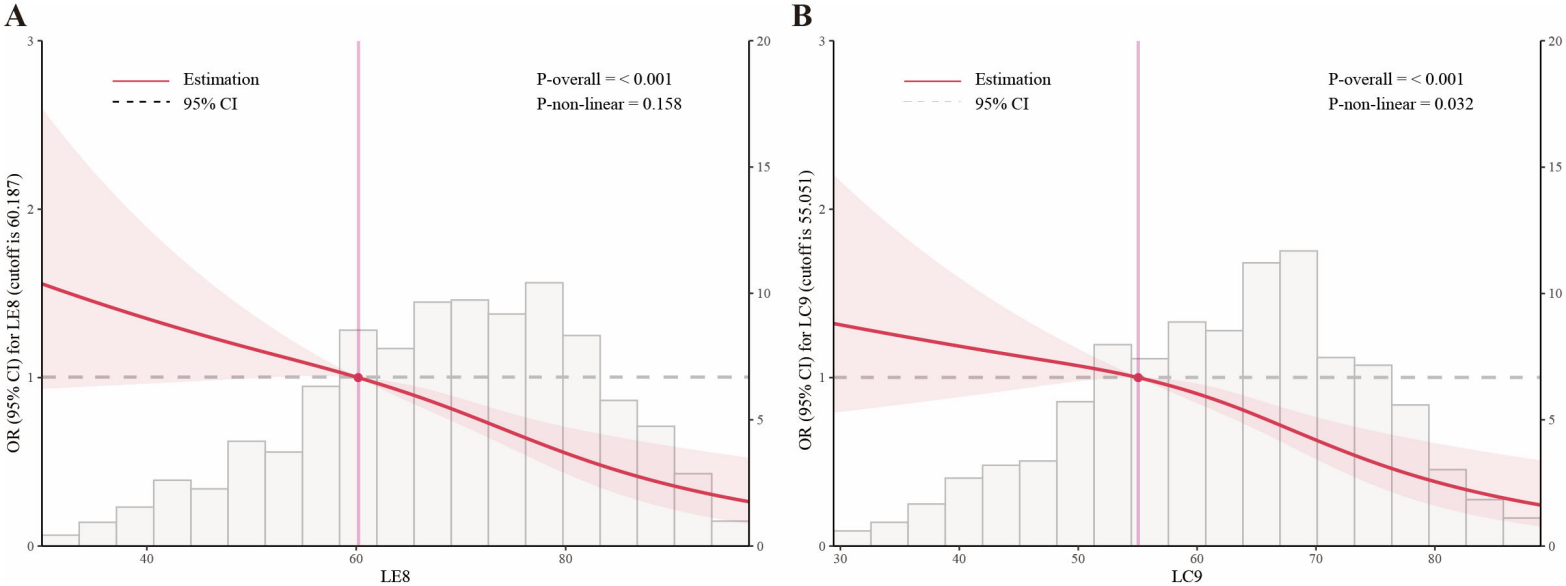
Association of LE8 **(A)** and LC9 **(B)** with infertility modeled using restricted cubic splines (RCS) in fully adjusted, survey−weighted logistic regression. Solid lines indicate adjusted odds ratios; shaded areas denote 95% CIs. RCS knots were placed at prespecified percentiles of the score distributions. The horizontal dashed line marks OR = 1.

Restricted cubic spline regression analyses (**Figure 3**) were conducted to assess potential nonlinear relationships between LE8, LC9, and infertility risk. The results demonstrated a significant nonlinear relationship, with a dose-dependent reduction in infertility risk as the LE8 and LC9 scores increased. The association was approximately linear up to a score of 60.187 for LE8 and 55.051 for LC9 (**Table 3**), beyond which the protective effect plateaued, suggesting a threshold effect. To further validate this pattern, we conducted sensitivity analyses stratified by tertiles of LE8 and LC9, confirming that women in the highest tertile had a significantly lower risk of infertility (p < 0.001). This analysis suggested that higher LE8 and LC9 scores are generally protective, but their effects may plateau at higher levels.

**Table 3:** Threshold effect analysis of LE8 and LC9 on infertility using a two-piecewise logistic regression model in adults in the NHANES 2013–2018.

Threshold effect analysis	Infertility OR (95%CI)	P-value
LE8		
Inflection point	60.187	
LE8 ≤ 60.187	0.99 (0.97, 1.01)	0.200
LE8 > 60.187	0.97 (0.95, 0.98)	**<0.001**
Log-likelihood ratio test		0.209
LC9		
Inflection point	55.051	
LC9 ≤ 55.051	0.99 (0.97, 1.01)	0.120
LC9 > 55.051	0.95 (0.92, 0.98)	**<0.001**
Log-likelihood ratio test		**0.032**

Model adjusted for age, race, education level, marital status, PIR, alcohol use.

Bold values indicates statistically significant results.

### Predictive modeling for infertility

**Figure 4:**
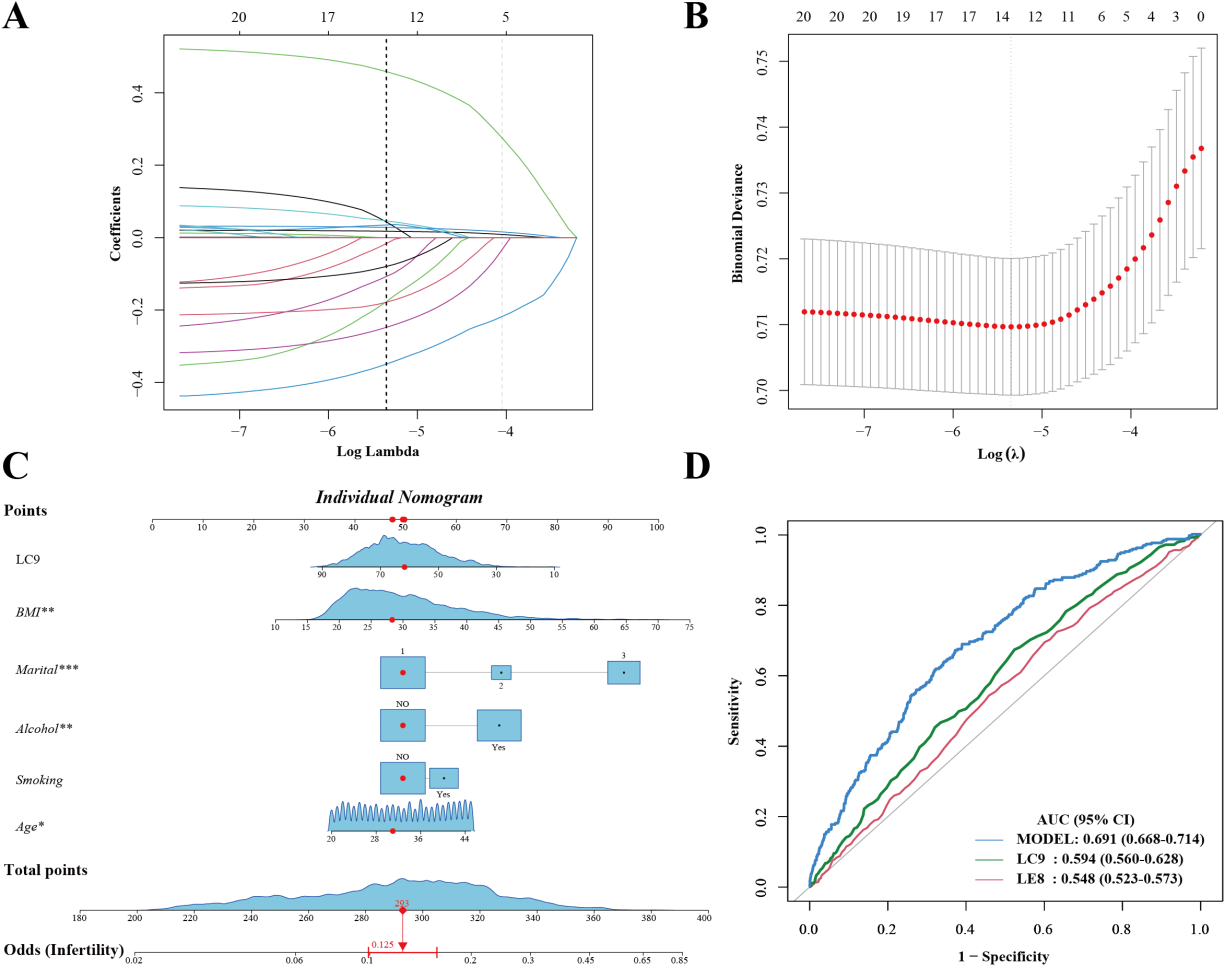
Development of a survey-weighted predictive nomogram for infertility using LASSO and logistic regression. **(A)** LASSO coefficient profiles. **(B)** Ten-fold cross-validation curve identifying the optimal λ. **(C)** Nomogram based on LASSO-selected predictors (LC9, age, BMI, marital status, smoking, alcohol). Red markers illustrate a sample patient’s inputs. **(D)** ROC curve of the final model (AUC 0.691, 95% CI 0.668–0.714). *Marital: 1.Married/Living with a partner; 2.Divorced/Separated/Widowed; 3.Never married.

LASSO regression was used to identify the most relevant predictors of infertility (**Figures 4A, B**). The final predictive model included LC9, BMI, marital status, smoking status, alcohol status, and age. A predictive nomogram was developed based on these selected variables (**Figure 4C**). The nomogram provided individualized risk prediction, where higher LC9 scores significantly reduced the estimated probability of infertility. The final model demonstrated moderate discrimination (AUC 0.691, 95% CI 0.668–0.714) (**Figure 4D**). Additional analyses of sensitivity, specificity, and predictive values were consistent with moderate discrimination. The calibration plots indicated good agreement between the predicted and observed probabilities of infertility risk (**Supplementary Figure S1**). The model provided limited net benefit within select threshold ranges in decision curve analysis (DCA) (**Supplementary Figure S2**). Compared with a base model including age, BMI, and smoking status, adding LE8 or LC9 modestly improved predictive accuracy, suggesting incremental value for risk stratification.

## Discussion

In the preceding Results section, our analyses demonstrated that higher LE8 and LC9 scores are significantly associated with a reduced risk of infertility in women of reproductive age. Notably, women with superior scores presented overall more favorable cardiometabolic profiles, consistent with mechanisms relevant to ovulatory function; however, direct measures of ovulatory patterns were not assessed in NHANES, and this finding was associated with improved metabolic and cardiovascular health markers. These findings support the concept that optimal cardiovascular and metabolic status creates a favorable environment for reproductive success. Marital status, smoking, and alcohol: The higher infertility prevalence among married/living−with−partner participants likely reflects greater exposure to attempting conception and reporting, rather than a causal effect of marriage per se. Smoking and alcohol were more common in the infertility group; both are biologically plausible risk factors via oxidative stress, endocrine disruption, and adverse vascular effects. In this discussion, we delve more deeply into the molecular and cellular mechanisms that underlie these associations.

A central component of this relationship is insulin resistance (IR) and its compensatory hyperinsulinemia (22). In an insulin-resistant state, target tissues—including the ovarian granulosa and theca cells—fail to respond adequately to insulin (23). This inadequacy prompts increased circulating insulin levels, which act on ovarian cells by upregulating key steroidogenic enzymes, such as the steroidogenic acute regulatory protein (StAR) and CYP17A1 (24). The resulting hyperandrogenism disrupts normal folliculogenesis, leading to arrested follicle development and anovulation (25). Moreover, high insulin levels suppress the hepatic production of sex hormone–binding globulin (SHBG), further increasing the bioavailability of androgens and aggravating the imbalance in the hypothalamic–pituitary–ovarian (HPO) axis (26, 27). These hormonal disturbances are central not only in the pathophysiology of PCOS but also in the broader context of infertility associated with metabolic dysfunction (28).

Chronic low-grade inflammation represents another pivotal mechanism linking poor cardiovascular health to infertility (16). Elevated levels of proinflammatory cytokines such as tumor necrosis factor-alpha (TNF-α) and interleukin-6 (IL-6) are common in individuals with metabolic syndrome (29). These cytokines impair insulin receptor signaling by promoting serine phosphorylation of insulin receptor substrate (IRS), thereby exacerbating IR (30). In the ovary, inflammatory cytokines directly affect granulosa cell function by reducing aromatase activity, which shifts the delicate balance between estrogen and androgens (31). This disruption compromises oocyte quality and the overall microenvironment required for successful follicular maturation and subsequent embryo development. In parallel, oxidative stress generated by excess reactive oxygen species (ROS) further fuels inflammation (32). ROS can damage mitochondrial DNA, lipids, and proteins within ovarian cells, and activate transcription factors such as NF-κB, which upregulate the expression of additional inflammatory mediators (33). The combined impact of these processes creates a vicious cycle that deteriorates both metabolic and reproductive functions.

Endothelial dysfunction is yet another factor that may mediate the relationship between cardiovascular health and infertility (34). Endothelial cells regulate vascular tone by producing nitric oxide (NO), a potent vasodilator essential for maintaining adequate blood flow to the ovaries and endometrium (35). In conditions characterized by poor cardiovascular health, such as hypertension and dyslipidemia, the reduced bioavailability of NO—often a consequence of oxidative stress—leads to impaired uterine perfusion (36). This diminished blood flow compromises the endometrial environment, reducing receptivity and interfering with embryo implantation.

Integrating psychological health into the LC9 score further refines our understanding of these interactions. Chronic psychological stress elevates cortisol levels, suppresses GnRH secretion from the hypothalamus and disrupts the pulsatile release of LH and FSH from the pituitary gland (19). This hormonal dysregulation can lead to irregular menstrual cycles and further impair ovulatory function. Moreover, sustained cortisol elevation contributes to systemic inflammation, thereby reinforcing the adverse effects on insulin sensitivity and endothelial function (37). Thus, incorporating mental health into LC9 emphasizes that stress management is crucial for maintaining reproductive capacity.

At the cellular level, these hormonal and inflammatory disturbances can lead to epigenetic modifications within ovarian and endometrial tissues (38). For example, oxidative stress and inflammation may alter DNA methylation patterns in granulosa cells, thereby affecting the expression of genes involved in follicular development and oocyte competence (39). Similarly, impaired glucose uptake due to reduced expression of GLUT4 in the endometrium may compromise cellular energy metabolism, further worsening endometrial receptivity (40). These molecular alterations decrease the quality of oocytes and impair the implantation process, increasing the risk of infertility. Furthermore, adipokines secreted by adipose tissue—such as leptin and adiponectin—play crucial roles in linking obesity, IR, and reproductive function (41). In obese states, elevated leptin levels, coupled with leptin resistance, may disrupt ovarian steroidogenesis and interfere with the normal regulation of the HPO axis (42). Conversely, reduced adiponectin levels diminish insulin sensitivity, exacerbating hyperinsulinemia and its downstream effects on androgen production and inflammatory pathways (41).

Overall, our findings underscore the multifactorial nature of infertility, where metabolic, inflammatory, vascular, and psychological factors converge to impair reproductive outcomes. The associations captured by the LE8 and LC9 scores reflect the cumulative burden of these interconnected mechanisms. Improving cardiovascular health through targeted lifestyle modifications—such as increased physical activity, improved dietary patterns, weight loss, stress reduction, and smoking cessation—can help mitigate insulin resistance, lower chronic inflammation, and restore endothelial function. These improvements benefit overall cardiovascular health and create a more favorable environment for ovarian function and embryo implantation.

By elucidating these detailed mechanisms, our study provides a rationale for integrating comprehensive assessments of cardiovascular and mental health into fertility evaluations. Future longitudinal studies that explore these pathways in greater detail will be essential for establishing causality and developing novel interventions that simultaneously address metabolic dysfunction and reproductive failure.

### Study strengths and limitations

This study has several notable strengths. First, it leverages a large, nationally representative dataset from the NHANES, thereby enhancing the generalizability of our findings to many women in the United States. The use of composite indices such as LE8 and LC9 provided a holistic view of cardiovascular, metabolic, and psychological health, elucidating the complex interplay between these factors and female reproductive function. Advanced statistical techniques—including multivariable regression, restricted cubic spline analyses, and extensive subgroup evaluations—were employed to assess the associations and potential dose–response relationships robustly. Additionally, our study provides an in-depth discussion of possible molecular mechanisms, including the roles of insulin resistance, chronic inflammation, oxidative stress, and endothelial dysfunction, that may help explain the observed associations between better cardiovascular health and improved fertility outcomes.

Despite these strengths, several limitations must be acknowledged. The cross-sectional design of the NHANES precludes any causal inferences, limiting our ability to determine the temporal sequence between improved cardiovascular health and fertility outcomes. Self-reported measures, such as infertility status, may be subject to recall bias and misclassification. Furthermore, although our models were adjusted for various potential confounders, residual confounding from unmeasured factors cannot be completely ruled out. Notably, the NHANES study population has certain limitations itself. The participants in the NHANES are U.S. residents who are generally noninstitutionalized and tend to have health insurance and relatively better access to healthcare, which may not reflect more diverse or underinsured populations. This inherent selection bias, along with the demographic characteristics of the NHANES participants, may limit the generalizability of our findings to other populations, including those from different countries or socioeconomic backgrounds. Finally, while our composite indices provide a broad health assessment, they may not capture the full spectrum of individual risk factors or the heterogeneity of the underlying pathophysiological mechanisms affecting fertility. Predictive performance was moderate (AUC ~ 0.69), with no external validation, which limits its immediate clinical utility. Quartile categorization may obscure linear trends; however, RCS analyses support near-linear reductions up to identified thresholds.

## Conclusion

Our study found that higher LE8 and LC9 scores were associated with a lower prevalence of self−reported infertility. While mechanisms are biologically plausible, the cross-sectional design, moderate AUC, and lack of external validation limit immediate clinical application. Prospective and interventional studies are needed to determine whether improving composite cardiovascular health may translate into better fertility outcomes.

## Data Availability

The original contributions presented in the study are included in the article/**Supplementary Material**. Further inquiries can be directed to the corresponding author.

## Supplementary material

Supplementary Figure 1 Calibration curves. Solid lines indicate bias-corrected predictions, gray dashed lines indicate apparent predictions, and black dashed lines indicate ideal predictions.

Supplementary Figure 2 Decision curve analysis (DCA). The blue line indicates the net benefit of the predictive model. The green line indicates that the predictive model was not used.
