# Supplementary figures and images for: Composite cardiovascular health indices (Life’s essential 8 and Life’s crucial 9) and female infertility: an NHANES 2013–2018 cross−sectional analysis
Source: Front Endocrinol (Lausanne). 2025 Nov 28;16:1581148. doi: 10.3389/fendo.2025.1581148 (PMC12698410; doi:10.3389/fendo.2025.1581148)

Actual Probability

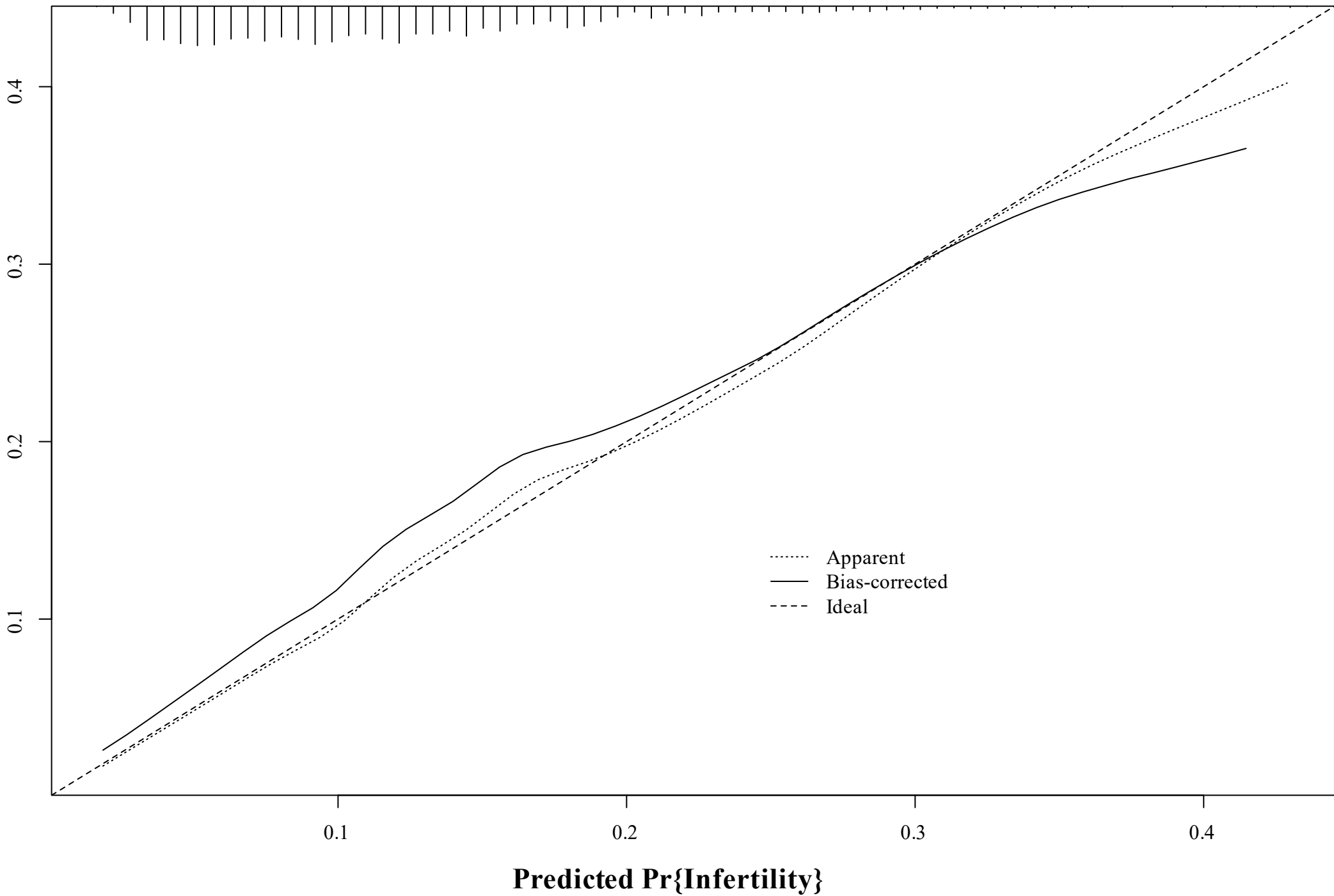

B= 1000 repetitions, boot

Mean absolute error=0.01 n=2360

Supplement: Supplementary Figure 1 — Calibration curves. Solid lines indicate bias-corrected predictions, gray dashed lines indicate apparent predictions, and black dashed lines indicate ideal predictions. [file Supplementaryfile1.pdf]

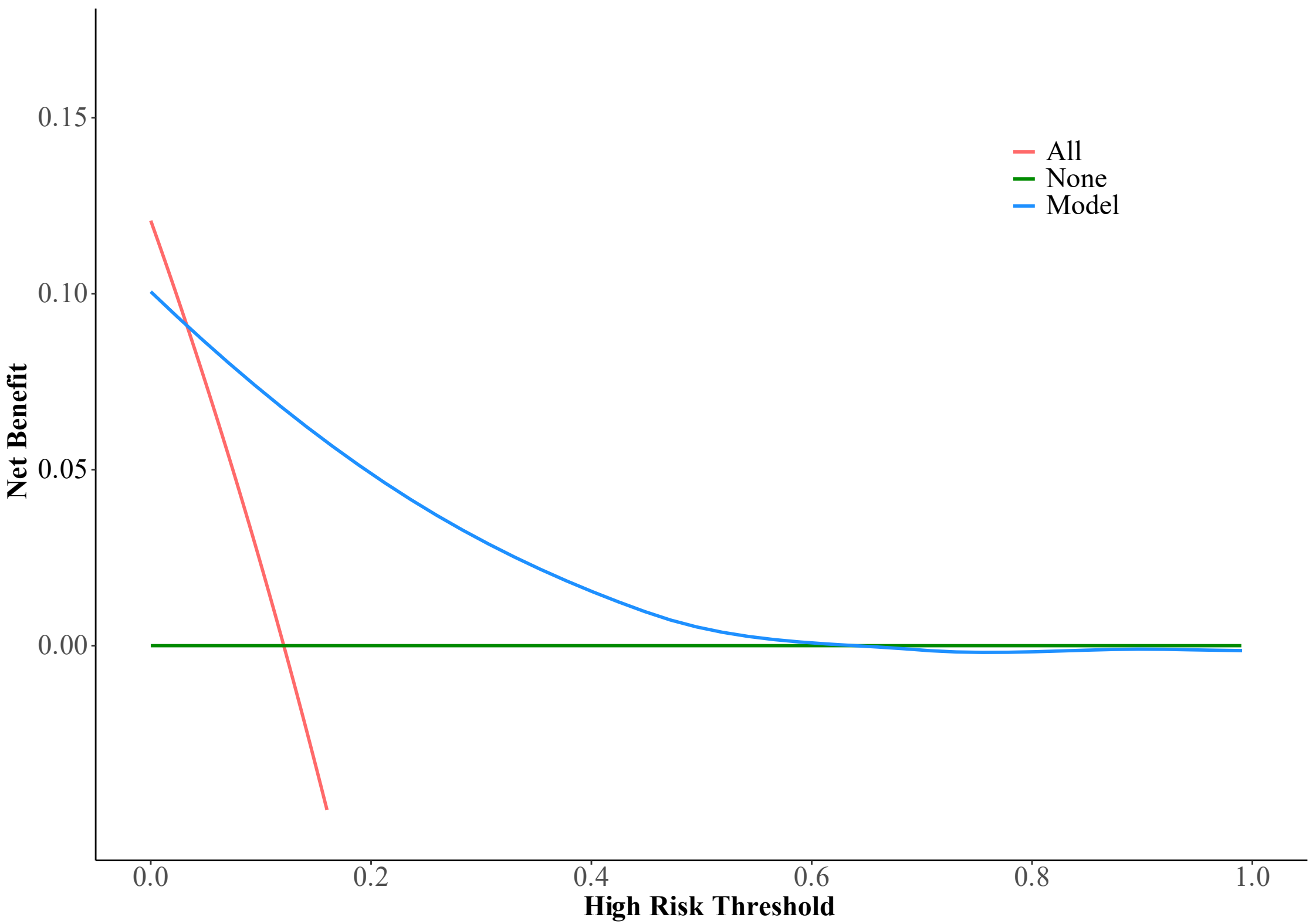

Supplement: Supplementary Figure 2 — Decision curve analysis (DCA). The blue line indicates the net benefit of the predictive model. The green line indicates that the predictive model was not used. [file Supplementaryfile2.pdf]
